# *Callyspongia* spp.: Secondary Metabolites, Pharmacological Activities, and Mechanisms

**DOI:** 10.3390/metabo13020217

**Published:** 2023-02-01

**Authors:** Yuni Elsa Hadisaputri, Annida Adha Nurhaniefah, Sendi Sukmara, Ade Zuhrotun, Rini Hendriani, Iyan Sopyan

**Affiliations:** 1 Department of Pharmaceutical Biology, Faculty of Pharmacy, Universitas Padjadjaran, Jatinangor 45363, Indonesia; 2 Department of Pharmacology and Clinical Pharmacy, Faculty of Pharmacy, Universitas Padjadjaran, Jatinangor 45363, Indonesia; 3 Departement of Pharmaceutics and Technology of Pharmacy, Faculty of Pharmacy, Universitas Padjadjaran, Jatinangor 45363, Indonesia

**Keywords:** *Callyspongia* spp., secondary metabolite, pharmacological activity, mechanism, cytotoxic

## Abstract

One of the most widespread biotas in the sea is the sponge. *Callyspongia* is a sponge genus found in the seas, making it easily available. In this review, the pharmacological activity and mechanism of action of the secondary metabolites of *Callyspongia* spp. are addressed, which may lead to the development of new drugs and targeted therapeutic approaches. Several scientific databases, such as Google Scholar, PubMed, ResearchGate, Science Direct, Springer Link, and Wiley Online Library, were mined to obtain relevant information. In the 41 articles reviewed, *Callyspongia* spp. was reported to possess pharmacological activities such as cytotoxicity against cancer cell lines (36%), antifungal (10%), anti-inflammatory (10%), immunomodulatory (10%), antidiabetic and antiobesity (6%), antimicrobial (8%), antioxidant (4%), antineurodegenerative (4%), antihypercholesterolemic (2%), antihypertensive (2%), antiparasitic (2%), antiallergic (2%), antiviral (2%), antiosteoporotic (2%), and antituberculosis (2%) activities. Of these, the antioxidant, antituberculosis, and anti-inflammatory activities of *Callyspongia* extract were weaker compared with that of the control drugs; however, other activities, particularly cytotoxicity, show promise, and the compounds responsible may be developed into new drugs.

## 1. Introduction

The ocean, which covers 71% of the earth’s surface, regulates our climate and contains abundant resources [1]. The sea encompasses a large area, but it is well connected, and the temperature is less extreme compared with that on land. Although containing more biodiversity, only 16% of all species have been identified [2].

One of the most ubiquitous sea organisms is the sponge. Sponges are often abundant in shallow water habitats, making them a unique biodiversity component [3]. They are one of the most diverse sessile organisms, with approximately 8876 valid species identified worldwide. Each has its unique characteristics, while some features are shared [4].

*Callyspongia* belongs to the family Callyspongidae. More than 60 species are widely distributed in the tropical sea [5]. It is also found in the Indian, Western Atlantic, and Eastern Pacific oceans, including Indonesia [6], the Red sea [7,8], Cuba [4], Barbados [9], Brazil [10,11], and Ecuador [12]. At a depth of 6–10 m below sea level, *Callyspongia* spp. can live under coral reefs, ranging from moderate to damaged conditions, or in habitats dominated by hard coral, sand, and coral rubble [13].

Sponges from the *Callyspongia* genus are formed from primary, secondary, and tertiary spongin fibers [4]. *Callyspongia* sponges are encrusting, form a single erect branch or a mass of round branches, and many are bifurcated. The longest branch that has been observed is approximately 40 cm. The branches are approximately 100–150 mm in diameter and have oscula or excretory organs that are slightly elevated, numerous, scattered throughout, and 0.5–2 mm in diameter. When pressed or cut, *Callyspongia* spp. secrete mucus. They have a smooth surface [14]. Skeletal fractions, such as spicules and cell debris, constitute 69.8% of the biomass of *Callyspongia* spp., the spongy cells (choanosomes) comprise 18.8%, and bacterial pellets account for 11.3%. The skeleton fraction dominates the biomass, resulting in the stiffer morphology of *Callyspongia* sponges [13].

**Figure 1:**
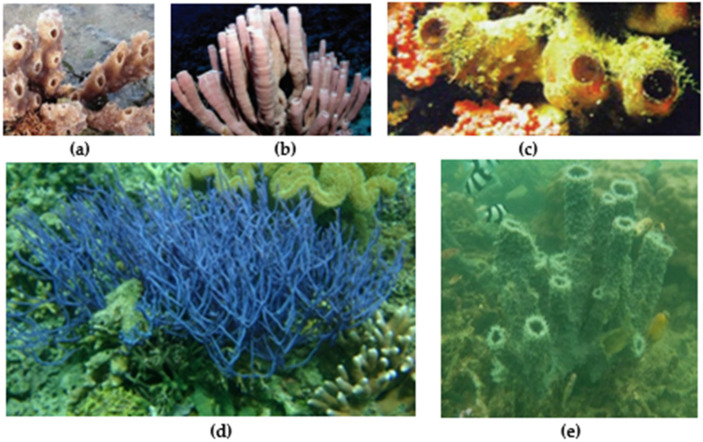
(**a**) *Callyspongia diffusa*, (**b**) *Callyspongia siphonella*, (**c**) *Callyspongia truncata* [15], (**d**) *Callyspongia samarensis* [3], (**e**) *Callyspongia aerizusa*.

The morphology of the callyspongia species varies. For example, *Callyspongia* (*Cladochalina*) *aerizusa* (Figure 1e) and *Callyspongia* (*Siphonochalina*) *siphonella* (Figure 1a) have a tubular and clustered form, but with different colors, tubes, and oscula size. *Callyspongia aerizusa* has a green–orange color, whereas *Callyspongia siphonella* has pale lavender color. There are also species with varying forms, such as *Callyspongia* (*Cladochalina*) *diffusa* (Figure 1b), which has a dull champagne pink or purplish-pink color, long or short thick cylindrical branches that vary from fanlike to upright or irregular [15]. Other species have unlikely forms, *Callyspongia samarensis* is called a spaghetti sponge because of its forms (Figure 1d) [16]. The color of *Callyspongia* spp. also varies with bright colors, such as *Callyspongia truncate* (Figure 1c), or deep colors like *Callyspongia aerizusa.*

Sponges are a potential repository of new drugs. There are several drugs originating from sponges that have entered clinical trials and approved, including cytarabine (Ara-C) for cancer treatment and vidarabine as an antiviral [17]. In addition, Eribulin mesylate (E7389) is an anticancer drug that is undergoing clinical phase 3 testing [18,19] Gemcitabine (GEM) (Gemzar) is an anticancer agent which has entered clinical phase 2 [20], whereas IPL576,092 (contignasterol derivative) is an anti-inflammatory compound that has entered clinical phase 2 testing [21]. PM-10450 (Zalypsis^®^) [22], discodermolide, HT1286 (hemiasterlin derivative), LAF389 (bengamide B derivative), hemiasterlin (E7974), KRN7000 (agelasphin derivative) [23], PM-060184 [24], and NVP-LAQ824 (psammaplin derivative) have entered clinical phase 1 trials as anticancer drugs [25].

Other pharmacological activities of sponge compounds include antibacterial, antihyperlipidemic, antiproliferative, immunomodulatory, and anti-inflammatory effects have been reported, including *Callyspongia* spp. [26,27]. Sponges contain multiple primary and secondary metabolites, such as fatty acids, alkaloids, steroids, nucleotides, peptides, polyacetylenes, and terpenoids. A total of 212 compounds have been isolated from *Callyspongia* spp. and their structures and bioactivities have been presented [28].

This review summarizes the potential pharmacological activities exhibited by *Callyspongia* spp. compounds that may be developed into new drugs. We also discuss the related mechanisms that may contribute to targeted therapy.

## 2. Materials and Methods

**Figure 2:**
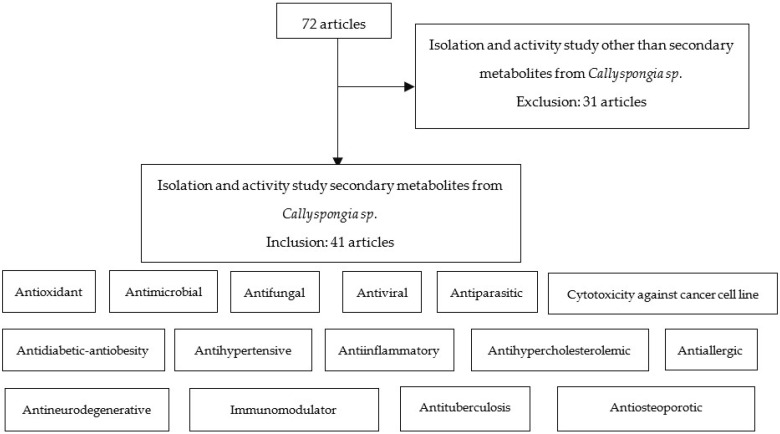
Method of screening information sources.

The literature review of *Callyspongia* spp. was based on topics related to pharmacological activity and the mechanism of action of secondary metabolites contained therein. This review was conducted with a qualitative and quantitative approach to obtain information from several scientific databases, including Google Scholar, PubMed, ResearchGate, Science Direct, Springer Link, and the Wiley Online Library. Several keywords, such as “*Callyspongia* sp.”, “metabolites”, and “pharmacology activity”, were used to procure relevant resources. The inclusion criterion for selecting articles was that they should describe the isolation and functional studies of secondary metabolites from *Callyspongia* sponges. Articles describing the isolation and activities of fungi or bacteria in *Callyspongia* species were excluded. The abstracts were carefully read to identify and select relevant articles. From 72 identified articles after screening information sources, 41 published between 1980 and 2021 were selected and reviewed (Figure 2).

## 3. Results

Sixteen pharmacological activities have been reported for *Callyspongia* spp. These activities along with their descriptions are listed in Table 1.

## 4. Discussion

We have discussed the pharmacological activities of *Callyspongia* spp. that have been previously reported.

### 4.1. Antidiabetic, Antihypercholesterolemic, and Antiobesity

**Figure 3:**
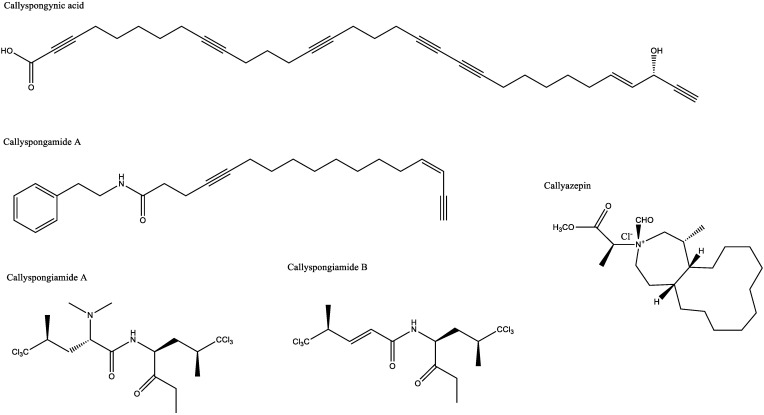

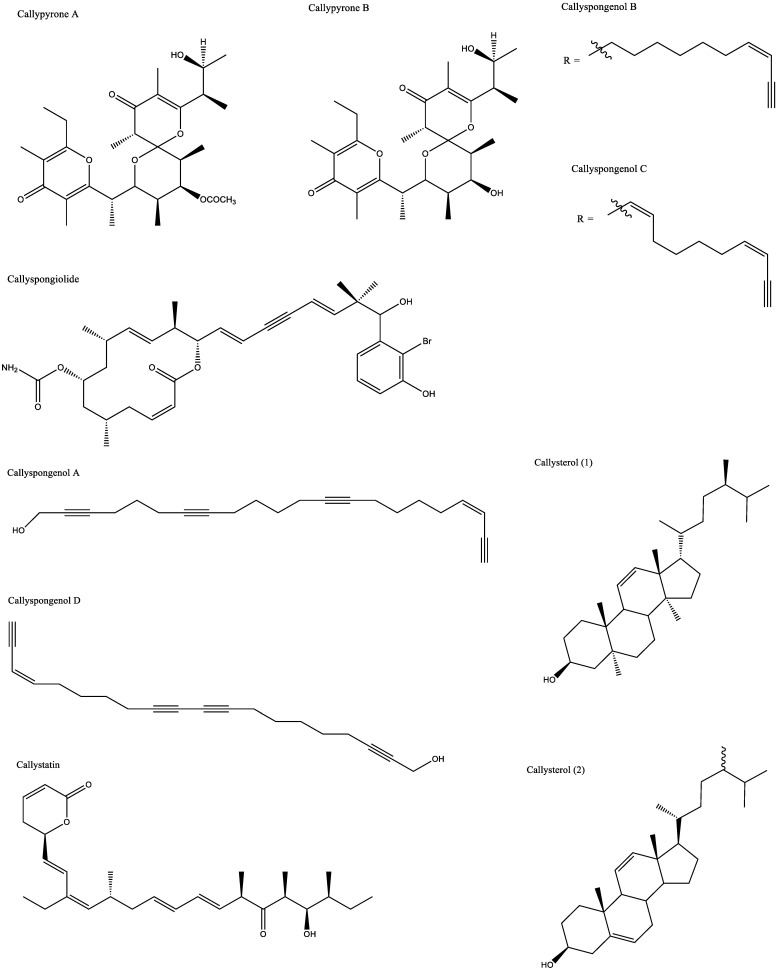

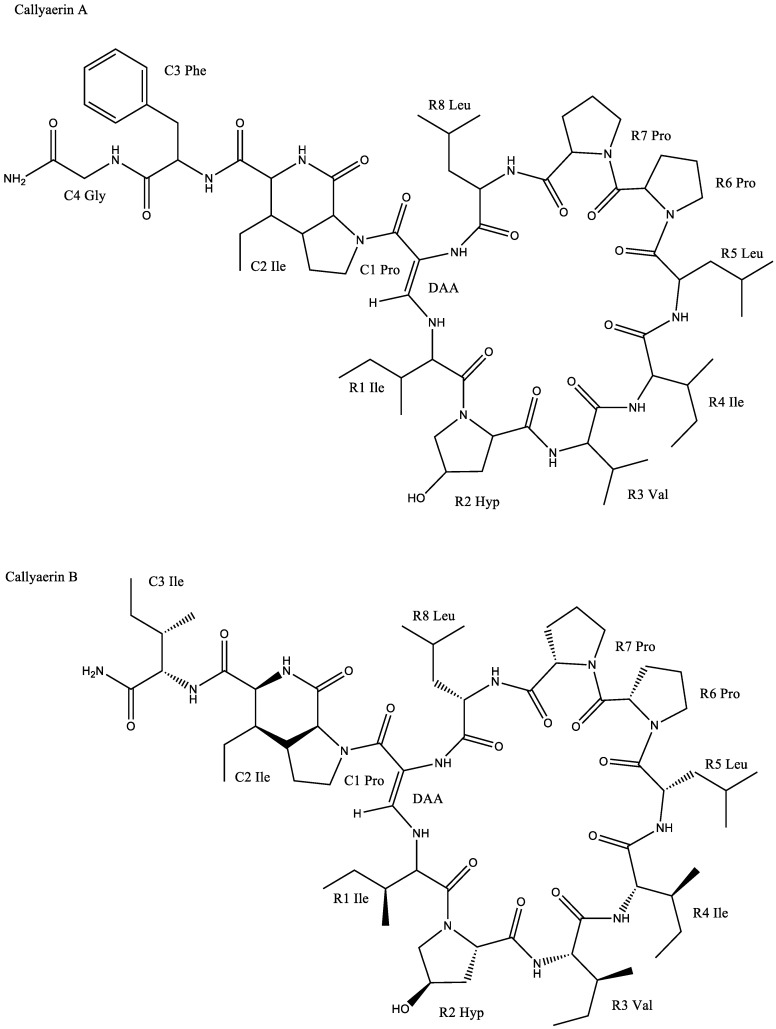

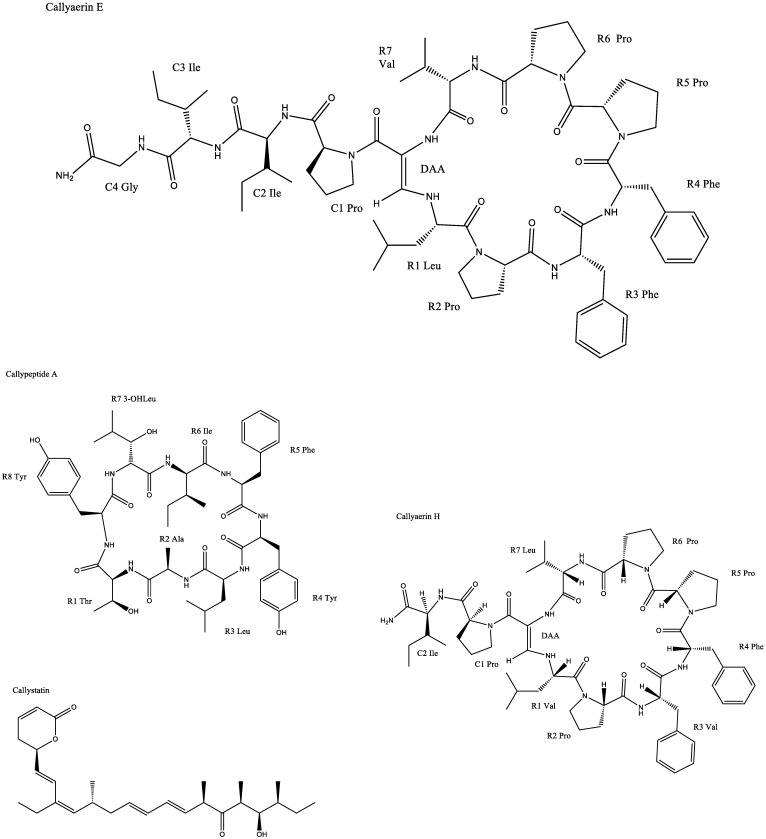
Structure of secondary metabolites from the *Callyspongia* subgenus.

The active compound from *Callyspongia truncata,* callyspongynic acid (Figure 3), shows higher antidiabetic activity by inhibiting α-glucosidase with an IC_50_ of 0.25 μg/mL [29,30] compared with acarbose (IC_50_ 1.3 μg/mL) [70]. Inhibiting this enzyme reduces caloric intake by attenuating appetite, suppressing hunger, and increasing satiety [71,72], thereby supporting weight loss [73] to a moderate level [74]. It is also one of the targets of diabetes therapy. Compared with α-amylase, inhibiting α-glucosidase can improve hyperglycemia, especially postprandial hyperglycemia, by decreasing glucose production (Figure 3) [75].

Compounds, such as callyspongiamide A and B as well as disamide A (Figure 3), exert antihypercholesterolemic activity, which can also lead to an antiobesity effect by inhibiting sterol O-acyltransferase (SOAT), the enzyme that catalyzes the formation of cholesteryl ester [76]. In addition, other sterols may be used as activators or substrates of this enzyme [77], which implicates it as a potential drug target [61] in hypercholesterolemia; however, the underlying mechanism remains unknown.

In a cell-based testing assay, the IC_50_ values of callyspongiamide A against SOAT 1 and SOAT 2 were 0.78 ± 0.19 and 2.8 ± 0.72 μM, those of callyspongiamide B were 1.2 ± 0.31 and 2.4 ± 0.96 μM, whereas those of disamide A were 5.2 ± 0.93 and 4.2 ± 0.76 μM. Although each compound markedly inhibited SOAT 2, as evidenced by the lower IC_50_ compared with the control beauveruolide III (IC_50_ > 20 μM), only callyspongiamide A significantly inhibited SOAT 1 [5].

**Figure 4:**
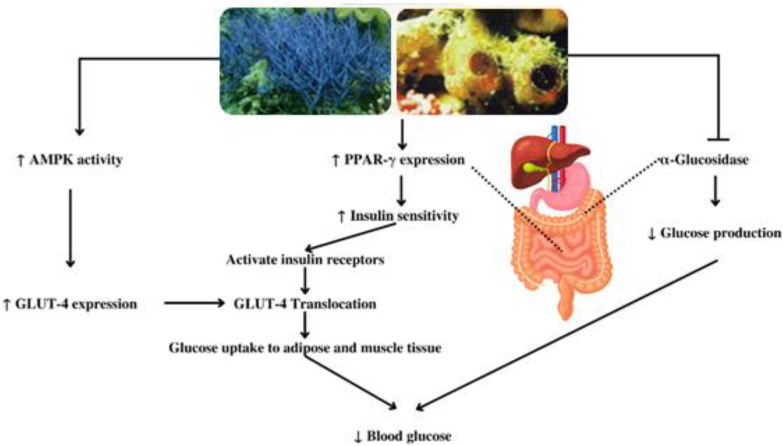
Antidiabetic mechanism of secondary metabolites from the subgenus *Callyspongia*.

*Callyspongia* sp. also contains β-Sitosterol. This compound exhibits potent antidiabetic activity related to insulin receptor activation and increased glucose transporter 4 (GLUT-4) translocation to adipose tissue [78,79]. In addition, these compounds can potentially maintain glucose homeostasis through sensitization of insulin resistance by increasing the expression of peroxisome proliferator-activated receptor and GLUT-4 (Figure 4) [80]. Another study on HFD-fed and sucrose-induced type-2 diabetic rats indicates that β-Sitosterol enhances the glycemic regulation [32,79].

The methanolic extract of *Callyspongia samarensis* also exerts antidiabetic activity by enhancing the activity of AMP-activated protein kinase (AMPK) with an EC_50_ of 14.47 μg/mL, which is more potent compared with the positive control aspirin (EC_50_ 100 μg/mL). This activity may originate from compounds with phenolic groups in the extract [31]. AMPK is an important target for treating type-2 diabetes because its activation affects various aspects of cellular metabolism. It increases glucose metabolism, uptake in the bone and muscle, fatty acid oxidation in the bone, muscle, and liver, mitochondrial oxidative capacity, and insulin sensitivity, whereas it decreases fatty acid synthesis in the liver through GLUT-4 expression (Figure 4) [81,82,83,84].

### 4.2. Antihypertensive

**Figure 5:**
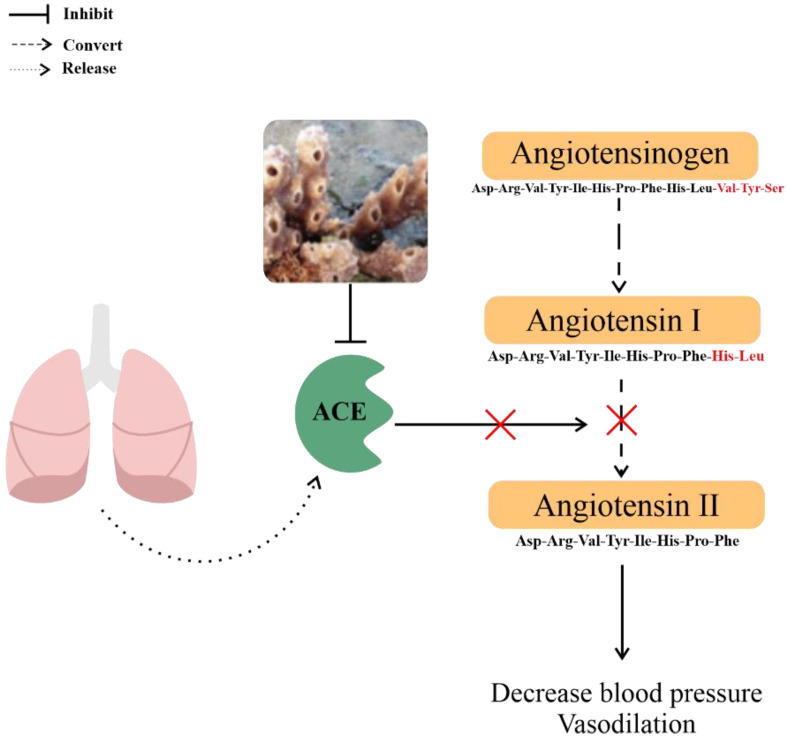
Antihypertensive mechanism of *Callyspongia diffusa*.

Callypyrone A and callypyrone B (Figure 3) from *Callyspongia diffusa* exhibit antihypertensive activity by inhibiting angiotensin I-converting enzyme (ACE), which leads to a reduction in angiotensin production. Because angiotensin can constrict blood vessels and increase the heart work rate [85], ACE inhibition results in vasodilation and a decrease in blood pressure (Figure 5). The IC_50_ values of these two compounds against ACE were 0.48 mM and 0.57 mM, respectively [33], weaker than the standard drug, captopril (IC_50_ 0.36 mM) [33]. From the results, Callypyrone A and callypyrone B are not considered antihypertensive.

### 4.3. Anti-Inflammation

**Figure 6:**
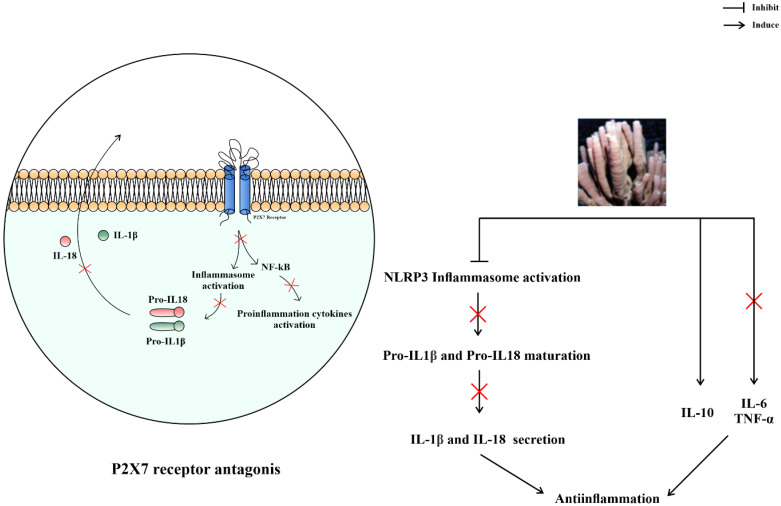
Prediction of anti-inflammatory mechanisms of compounds from the subgenus *Callyspongia*.

Diketopiperazines derived from *Callyspongia* sp., such as cyclo(L-Hyp-L-Ala), cyclo(L-Pro-Gly), cyclo(L-Pro-Phe), and cyclo(L-Pro-Ala), at a concentration of 5 µg/mL, showed anti-inflammatory activity by increasing the secretion of the anti-inflammatory cytokine, interleukin-10 (IL-10) (Figure 6). IL10 levels were increased by 1.65-, 1.29-, 1.54, and 1.56-fold in J774A.1 cells, respectively [36].

The anti-inflammatory activity of β-Sitosterol is independent of the adrenal pituitary axis. It inhibits the maturation of IL-1β via the NOD-, LRR-, and pyrin domain-containing protein 3 (NLRP3) inflammasome and inhibits other inflammatory cytokines, such as IL-6 and tumor necrosis factor-α [86]. In a carrageenin-induced edema model in bilaterally adrenalectomized rats, β-Sitosterol exhibited a 54% anti-inflammatory effect at a dose of 320 mg/kg, which was weaker than the control, oxyphenbutazone, which had a 74% anti-inflammatory effect at a dose of 100 mg/kg [37]. Therefore, β-Sitosterol has no potential as an anti-inflammatory agent.

**Figure 7:**
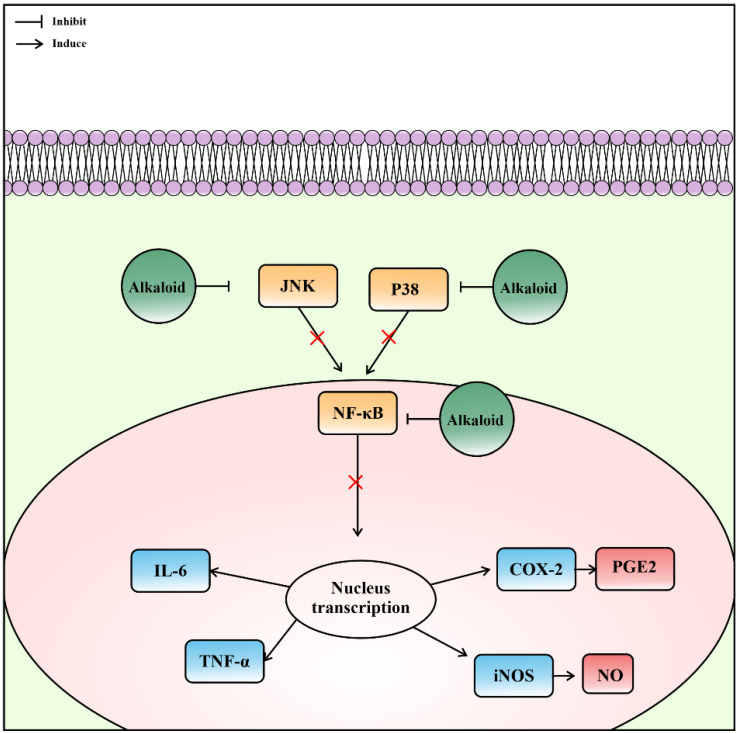
Prediction of anti-inflammatory mechanisms of alkaloids from the subgenus *Callyspongia*.

Niphatoxin C significantly affects the viability of pre-monocytic THP 1 cells, which express the P2X7 receptor [65]. Activation of this receptor promotes inflammation by releasing inflammatory cytokines, such as IL-18 and IL-1β, and by activating the NLRP3 inflammasome [87,88]. Thus, an antagonist of this receptor may inhibit the secretion of these cytokines (Figure 7). Furthermore, it can inhibit allograft rejection [87], sterile liver inflammation [88], and can potentially treat inflammatory diseases, such as osteoarthritis, rheumatoid arthritis, and chronic obstructive pulmonary disease [28,37].

Callysterol (Figure 3) from *Callyspongia siphonella* exhibits anti-inflammatory activity against rat paw edema that was similar to the control drug, cortisone. The activity was measured by a reduction in edema volume of 19.5 ± 7.3 mL for callysterol and 17.0 ± 7.0 mL for cortisone, whereas the negative control was 61.9 ± 4.7 mL [38]. *Callyspongia crassa* extracts also showed anti-inflammatory effects with a 61.47% inhibition of protein denaturation [34]. Alkaloids are considered responsible for these anti-inflammatory mechanisms [89], which vary according to the metabolite. The alkaloid that has been identified from ethanolic extract of *Callyspongia siphonella* was 5-bromo trisindoline and 6-bromo trisindoline [7]. Although the specific mechanism of 5-bromo trisindoline and 6-bromo trisindoline is unknown, some indole alkaloids were known to interfere with the nuclear factor-κB and c-Jun N-terminal kinase signaling pathways [90,91], preventing the synthesis or action of specific pro-inflammatory cytokines, and suppressing histamine release and nitric oxide production (Figure 7) [92,93]. Alkaloids are effective for treating inflammatory bowel disease [94,95,96,97].

### 4.4. Antifungal

Callyaerin A, B, and E (Figure 3) from *Callyspongia aerizusa* were shown to potently inhibit *Candida albicans*, with zones of inhibition of 25–30 mm, 15 mm, and 20 mm, respectively using the same concentration. Callyaerin A and E were more potent than callyaerin B [6].

Gelliusterol E from *Callyspongia* aff. *implexa* also exerts activity against chlamydial fungi in a dose-dependent manner by inhibiting the formation and growth of chlamydial inclusions. At the highest concentration tested (40 μM), no inclusions were observed, similar to the effect of the control, tetracycline. Thus, this compound not only inhibits the formation of *Chlamydia,* but also affects its development cycle [39]. In addition, the structure of gelliusterol E is similar to that of cholesterol, which is needed for the growth of *Chlamydia trachomatis*. Furthermore, this compound inhibits lipid acquisition and fungal growth [98].

β-Sitosterol compounds found in *Callyspongia* sp. also exhibit antifungal activity against *Fusarium* spp, with 10 mm of average inhibition diameter [39,40]. (-)-siphonodiol from sponges display antifungal activities against *Trichophyton asteroids*, with moderately strong activity (MIC 25.0 μg/mL) [41]. Meanwhile, 4-hydroxybenzoic acid against *Ganoderma boninense* and (−)-loliolide display a broad spectrum of activity [42,43]. Active secondary metabolites that attack fungi are responsible for these antifungal activities, but their specific mechanisms of action remain unclear [99].

### 4.5. Cytotoxicity against Cancer Cell Lines

*Callyspongia siphonella* and *Callyspongia crassa* crude extracts were cytotoxic against a colon cancer (Caco-2) cell line with IC_50_ values of 5.57 μg/mL and 13.05 μg/mL, respectively, and against breast cancer (MCF-7) cell line with IC_50_ values of 1.39 μg/mL and 9.47 μg/mL [34]. Neviotine-C, neviotine A, sipholenol-A, and sipholenol from *Callyspongia siphonella* also exhibited cytotoxicity against cancer cell lines (Table 1). Sipholenol-A showed higher activity against the PC-3 and A549 cell lines (IC_50_: 7.9 ± 0.12 μM and 8.9 ± 0.01 μM), sipholenol L against the HepG-2 cell line (IC_50_: 18.7 ± 0.9 μg/mL), and sipholenone A against the MCF-7 cell line (IC_50_: 36.2 ± 0.13 μM or 3 ± 0.4 μg/mL) [44,45].

Callyspongiolide, extracted from *Callyspongia* sp., exhibited an IC_50_ of 320 nM against a mouse lymphoma cell line (L5178Y), 70 nM against human Jurkat J16 T cells, and 60 nM against Ramos B lymphocyte cells [46]. Callypeptide A (Figure 3) inhibited the growth of human cancer cells with GI_50_ values of 29 μM against breast adenocarcinoma (MDA-MB-231), 30 μM against colorectal carcinoma (HT-29), and 18.5 μM against lung carcinoma (A549). Its activity was weaker compared with doxorubicin as a control (GI_50_ values of 0.30, 0.40, and 0.35 μM, respectively) [47].

Callyazepine and (3R)-methylazacyclodecane (Figure 3) exhibited IC_50_ values of 7.4 μM and 3.2 μM against K562 cells, and 3.0 μM and 3.8 μM against A549 cells, respectively [100]. Hysmenialdisine from *Callyspongia* sp. had an IC_50_ value of 3.1 μM against colonic adenocarcinoma cells (SW620) and 2.0 μM against epidermoid carcinoma cells (KB-3-1) [49]. In addition, it produces akaterpine, which exhibited an IC_50_ of 0.5 μg/mL against phosphoinositide-specific phospholipase C [58]. Callyspongamide A (Figure 3), isolated from *Callyspongia fistularis*, had an IC_50_ of 4.1 μg/mL against HeLa cells [55].

**Figure 8:**
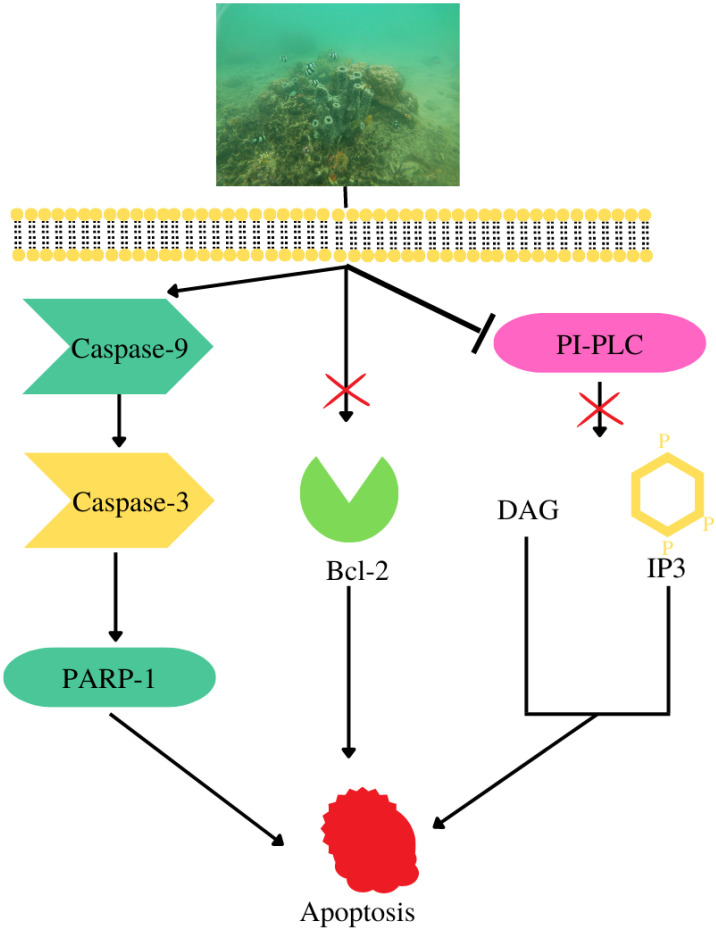
Cell death mechanisms of *Callyspongia aerizusa* compounds.

Methanolic extract of *Callyspongia aerizusa* had IC_50_ values of 9.38 μg/mL against A549 cells, 3.12 μg/mL against TE-8 cells, 10.62 μg/mL against HepG-2 cells, and 10.72 μg/mL against MIA PaCa-2 cells [59]. It also produces callyaerin E and H (Figure 6), which exhibited IC_50_ values of 0.39 μM and 0.48 μM against L5178Y cells [6]. *Callyspongia aerizusa* extract stimulates the expression of caspase-9, which in turn activates caspase-3, and subsequently downregulates Bcl-2, a key regulator of antiapoptosis (Figure 8) [59].

Callystatin (Figure 3) from *Callyspongia truncata* exhibited IC_50_ values of 0.01 μg/mL against KB cells and 20 pg/mL against L1210 cells [101]. *Callyspongia* sp. also contains two unknown compounds with antiproliferative activity against TR-LE cells: (−)-(3R,18R) alcohol with an IC_50_ of 0.11 μM and (+)-(3S,18S) with an IC_50_ of 0.47 μM [52].

The US National Cancer Institute classifies the cytotoxicity of a compound as high if its IC_50_ < 20 μg/mL, moderate if it falls between 21–200 μg/mL, weak if it falls between 201–500 μg/mL, and non-cytotoxic if the IC_50_ > 500 μg/mL [102]. Based on these criteria, most *Callyspongia* extracts possess high cytotoxic activity, except that of *Callyspongia schulzei*, which exhibits moderate activity, but no study was conducted against non-cancerous cell line except for *Callyspongia aerizusa* extract. Cytotoxicity of the methanol extract of *Callyspongia aerizusa* against TE-8 cells (IC_50_ 3.12 μg/mL) was more effective compared with that of the control drug, cisplatin (IC_50_ 8.1 μg/mL/27 μM), meanwhile this extract was non-cytotoxic for non-cancerous cell (HET-1A cell) up to 1000 μg/mL. The compounds responsible for suppressing A549 cell proliferation were identified as ergots-22-en-3-one and ergost-7-en-3-ol [59,103].

### 4.6. Antimicrobial and Antiparasitic

**Figure 9:**
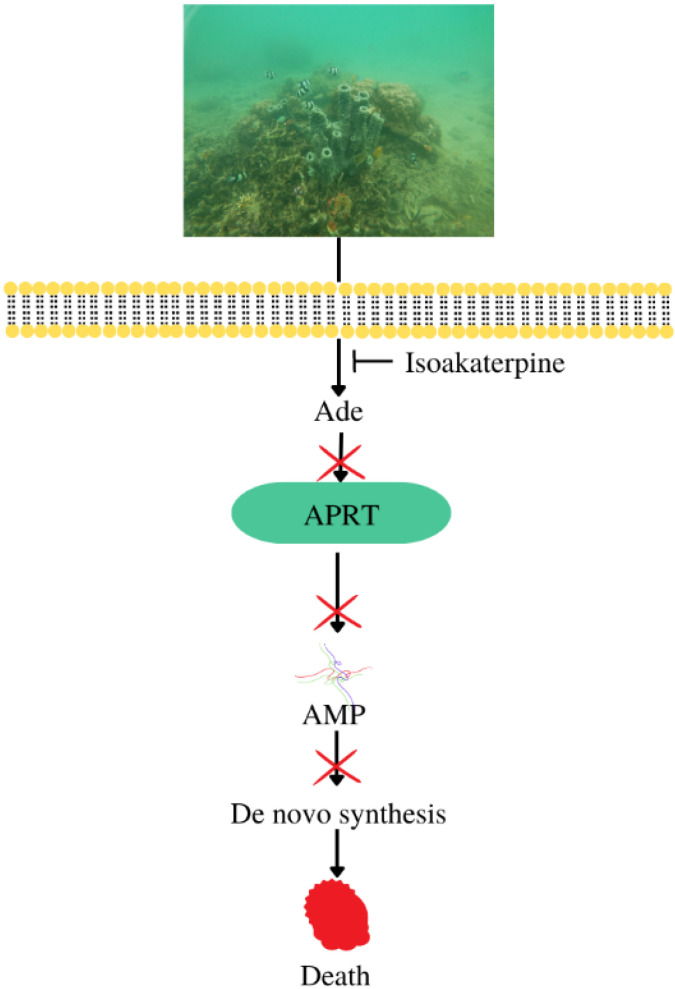
Antiparasitic mechanism of compounds from *Callyspongia* sp.

Isoakaterpine compounds from *Callyspongia* sp. exert antiparasitic activity by inhibiting adenosine phosphoribosyltransferase, one of the functional routes in *Leishmania* adenine metabolism, with an IC_50_ of 1.05 μM [11,104], resulting in death of the parasite (Figure 9).

Besides antiparasitic activity, the subgenus *Callyspongia* possesses antituberculosis activity resulting from callyaerins A and B (Figure 5) isolated from *Callyspongia aerizusa*. Their MIC_90_ values (2 μM and 5 μM, respectively) were less effective compared with the controls, rifampicin (<1 μM), ethambutol (1.25 μM), and isoniazid (0.625 μM). Beside the weaker activity compared with the control, there is no in vivo data to support this activity. These compounds inhibited the growth of *Mycobacterium tuberculosis* as evidenced by reduced cell viability using the resazurin dye reduction method and measuring cell fluorescence [105].

Siphonocolin from *Callyspongia siphonella* exhibited antimicrobial activity against *Pseudomonas aeruginosa* with an MIC of 64 μg/mL [61]. Moreover, neviotine A, sipholenol L, and sipholenone A from *Callyspongia siphonella* also exhibited antimicrobial activity against *Staphylococcus aureus*, *Bacillus subtilis*, and *Escherichia coli* (Table 1). Neviotine has higher antimicrobial activity with a zone of inhibition against *Staphylococcus aureus* of 14.1 ± 0.72 mm, against *Bacillus subtilis* of 17.2 ± 0.58 mm, and against *Escherichia coli* of 12.7 ± 0.58 mm [45].

*Callyspongia crassa* extract potently inhibited *Bacillus subtilis* and *Staphylococcus aureus* with zones of inhibition of 16–25 mm (at concentration 500 μg/mL), 9–15 mm and 16–25 mm (at concentration 250 μg/mL) respectively, while exhibiting high activity against marine bacteria. The IC_50_ of the extract was determined by a microdilution test and ranged from 5 μg/mL to 500 μg/mL. *Callyspongia crassa* is the most active among the Red sea sponges against *Bacillus subtilis*, with an LC_50_ 18.2 ± 3.56 μg/mL, but was weak against *Staphylococcus aureus* with an LC_50_ 215.2 ± 32.9 μg/mL [60]. Callyaerin A also exhibits antimicrobial activity against *Escherichia coli* and *Staphylococcus aureus*, with zones of inhibition of 10–15 mm and 9 mm, respectively, whereas callyaerin E has activity against *Escherichia coli*, *Bacillus subtilis*, and *Staphylococcus aureus*, with zones of inhibition of 9–11 mm, 15–17 mm, and 9–10 mm, respectively [6].

### 4.7. Antioxidant

Sponge extracts inhibited oxidative stress and carbohydrate hydrolysis enzymes linearly in a dose-dependent manner. Based on the 2,2-diphenyl-1-picryl-hydrazyl-hydrate assay, a *Callyspongia aerizusa* extract displayed an antioxidant activity of 56.6% at 0.5 μg/mL, 57.2% at 0.6 μg/mL, and 58.4% at 0.7 μg/mL, indicating that it may be classified as an antioxidant (>50%). *Callyspongia crassa* extract showed an antioxidant activity of 58.1% at 671 μg/mL, which was lower than the control, ascorbic acid (>90%), likely because *Callyspongia* was used in the form of an extract [34,62].

### 4.8. Antiallergic

The compound 3-(2-(4-hydroxyphenyl)-2-oxoethyl)-5,6-dihydropyridine-2(1H)-one was isolated from an ethanol extract of *Callyspongia* sp. [63]. This δ-lactam derivative was predicted to possess antiallergic activity based on its in silico inhibition of β-hexosaminidase (β-hex), which was determined using Origin 8.0. This compound inhibited β-hex activity in rat basophilic leukemia cells (RBL-2H3) with an IC_50_ of 18.7 ± 6.7 μM, which was weaker than the positive control, ketotifen fumarate (IC_50_ 15.0 ± 1.3 μM), but more potent than azelastine (IC_50_ 32.0 μM) [64]. β-hex is released from mast cell degranulation, thus its activity can be used as a biomarker of mast cell allergic response to quantify degranulation [106,107,108].

### 4.9. Antiviral

*Callyspongia crassa* and *Callyspongia siphonella* extracts exhibited cytotoxic effects on Vero cells, which were cultured for the isolation and multiplication of enterovirus and hepatitis A virus, with MICs of 9.765 μg/mL and 0.625 μg/mL, respectively. The maximum non-toxic concentrations of these extracts were 4.9 and 0.3 μg/mL, respectively. *Callyspongia crassa* crude extract had an antiviral activity of 85.3%, whereas the antiviral activity of *Callyspongia siphonella* extract was 16.4% [34].

### 4.10. Immunomodulatory

**Figure 10:**
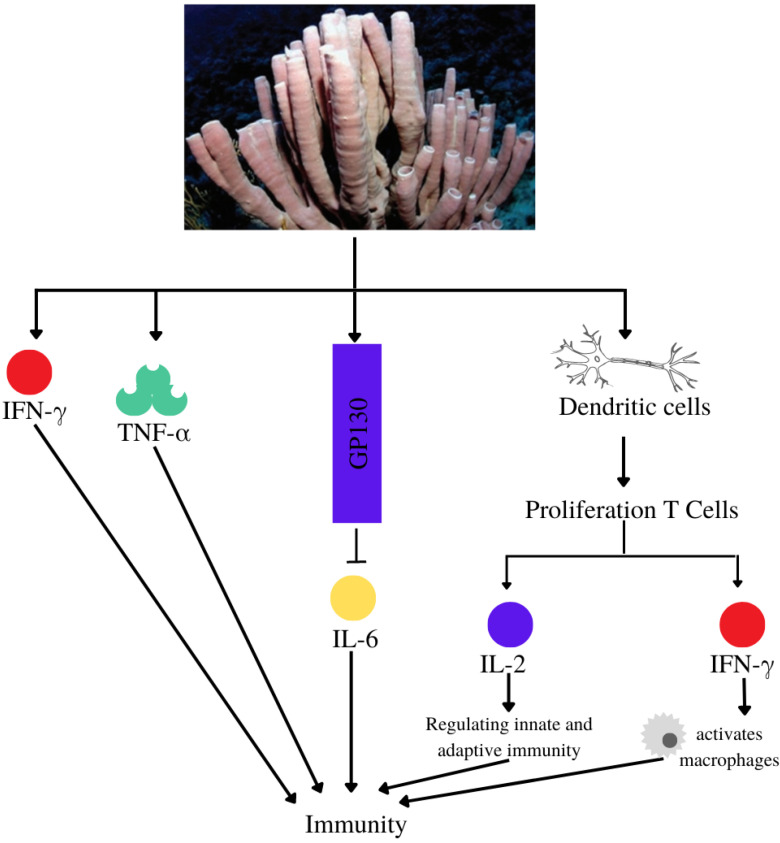
Immunomodulatory mechanisms of compounds from the subgenus *Callyspongia*.

*Callyspongia* extract at doses of 300 mg/kg and 400 mg/kg body weight, increased *S. aureus*-induced production of interferon-γ (IFN-γ) (455.265 pg/mL and 384.319 pg/mL) and tumor necrosis factor-α (TNF-α)(954 pg/mL and 1042 pg/mL) in male Wistar rats. It was more effective compared with 0.5% carboxymethyl cellulose sodium as negative control (160.314 pg/mL for INF-γ and 785.5 pg/mL for TNF-α) and bay leaf extract as positive control (353.486 pg/mL for INF-γ and 976 pg/mL for TNF-α) [68]. β-Sitosterol compounds from *Callyspongia* spp. modulate the activity of dendritic cells and increase the viability of peripheral blood mononuclear cells [67]. Siphonodiol, callyspongidiol, and 14,15-dihydrosphonodiol modulate the function of dendritic cells for T1 cell proliferation as well as IL-2 and IFN-γ production [66]. IL-2, along with other ILs, regulates innate and adaptive immunity by promoting an increase in the population of various immune cells [109,110]. Meanwhile, IFN-γ activates macrophages and enhances their immune response [111]. *Callyspongia* extract can stimulate the branch of the immune system involved in forming a receptor complex with gp130 to eventually inhibit the bioactivity of IL-6 (Figure 10) [112,113].

### 4.11. Antineurodegenerative

- β-secretase 1

Selectively inhibiting β-secretase 1 in specific subcellular compartments is an effective strategy to reduce the accumulation of neurotoxic amyloid plaques [114]. The methanol extract of *Callyspongia samarensis* significantly and non-competitively inhibited β-secretase 1 (IC_50_ 99.82 μg/mL). An acute oral toxicity test revealed that the extract was non-toxic, with an LD_50_ value of less than 2000 mg/kg. Moreover, an unknown compound in the extract, with a mass/charge ratio of 337.9 [M + H]+, was able to permeate the blood–brain barrier, making it a suitable candidate for developing central nervous system drugs [31].

- Kinase inhibitor

**Figure 11:**
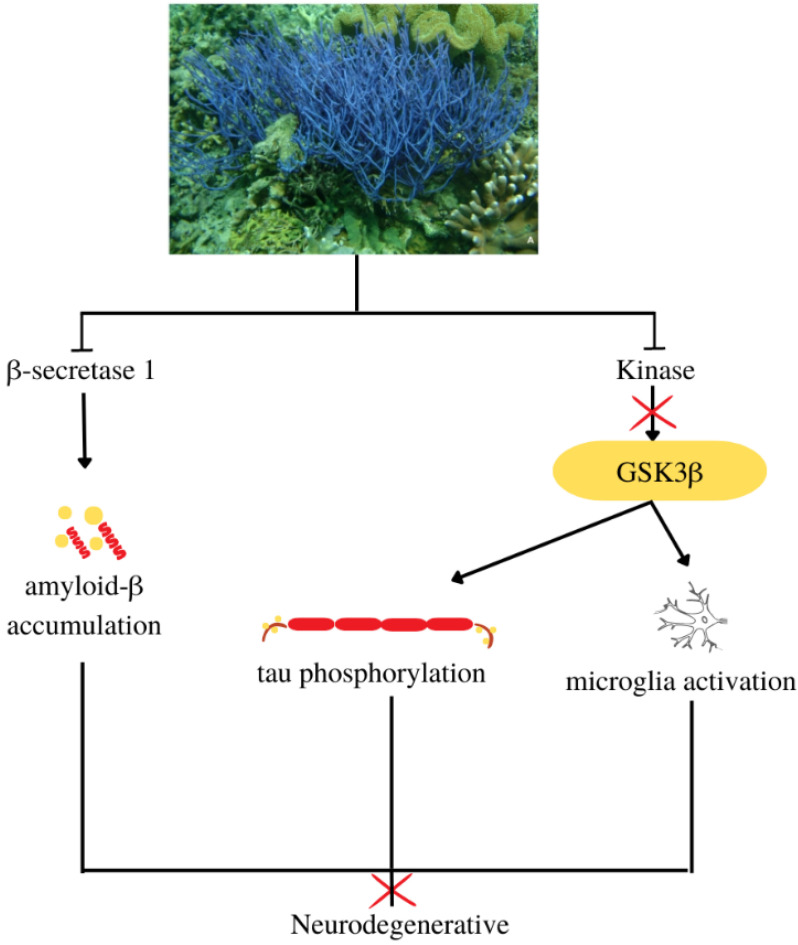
Antineurodegenerative mechanisms of compounds from the subgenus *Callyspongia*.

Kinases have a role in neurodevelopmental and central nervous system physiology. Activation of the glycogen synthase kinase 3β (GSK3β) results in tau phosphorylation, amyloid-β accumulation, microglia activation, neurogenesis, and memory abnormalities [115]. This suggests that its inhibition restores and repairs pathways and neurogenesis (Figure 11) [116,117]. Hymenialdisine, isolated from *Callyspongia* sp. (CMB-01152), inhibits casein kinase 1, cyclin-dependent kinase 5, and GSK3β with IC_50_ values of 0.03 μg/mL, 0.16 μg/mL, and 0.07 μg/mL, respectively. They abnormal hyperphosphorylate highly soluble microtubule-associated proteins to produce neurofibrillary tangles [49].

### 4.12. Antiosteoporotic

**Figure 12:**
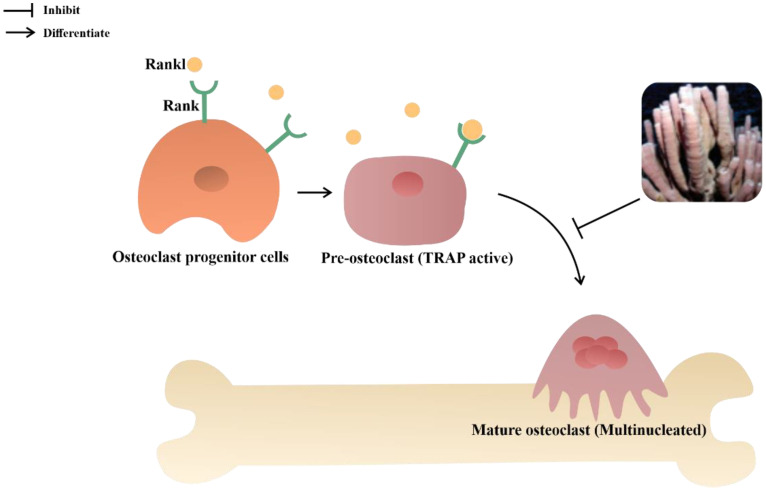
Antiosteoporotic mechanisms of Neviotine A and D from the *Callyspongia siphonella*.

Neviotine A and D are isolated triterpene-type compounds from *Callyspongia siphonella*. These compounds possess antiosteoporotic activity by inhibiting receptor activator of nuclear factor-kB ligand (Rankl) with IC_50_ values of 32.8 μM and 12.8 μM (quercetin as positive control: 25 μg/mL) [69]. The interaction between Rankl and Rank receptor translocate the tumor necrosis factor receptor-associated factors (TRAF6) to the RANK cytoplasmic domain, results in the activation of ERK, p38, and JNK via activation of signaling cascades and downstream targets. Thus, AP-1 and NF-kB transcription factors were activated and stimulated the formation and activity of osteoclasts, which affect resorption activity [118]. Neviotine A and D inhibit cell differentiation into multinucleated tartrate-resistant acid phosphatase (TRAP)-positive osteoclasts, which was upregulated via RANKL-induced osteoclastogenesis (Figure 12) [69,119].

## 5. Conclusions

**Figure 13:**
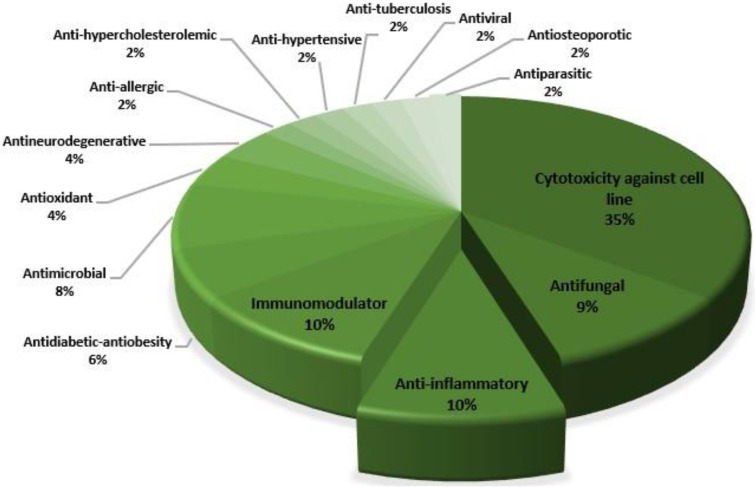
Distribution of the pharmacological activities reported in *Callyspongia* spp.

In the 41 articles we reviewed, the pharmacological activities that *Callyspongia* spp. is reported to possess include cytotoxic against cancer cell line (36%), antifungal (10%), anti-inflammatory (10%), immunomodulatory (10%), antidiabetic and antiobesity (6%), antimicrobial (8%), antioxidant (4%), antineurodegenerative (4%), antihypercholesterolemic (2%), antihypertensive (2%), antiparasitic (2%), antiallergic (2%), antiviral (2%), antiosteoporotic (2%), and antituberculosis (2%) activities (Figure 13). The most studied pharmacological activity is cytotoxicity against cancer cell lines. Most of the research was limited to in vitro testing and there is insufficient in vivo data to support such activity. In addition, not all secondary metabolites responsible for certain activities have been identified. Several activities require modification and further study because of a lack of testing or low activity. For example, the antiallergic activity of *Callyspongia* sp. predicted from in silico results or the antioxidant, antituberculosis, and anti-inflammatory activities of *Callyspongia* extract were weaker compared with those of the control drugs. Although many promising compounds with a high potential to become drugs remain to be comprehensively evaluated in vivo, *Callyspongia* with its known mechanisms of action, such as antidiabetic and cytotoxic effects, may be further developed for targeted therapy.

## Figures and Tables

**Table 1:** Pharmacological activities of *Callyspongia* spp.

Pharmacological Activity	*Callyspongia* spp.	Secondary Metabolite	Description of Activity	Ref.
Antidiabetic and antiobesity	*Callyspongia* *truncata*	Callyspongynic acid	IC_50_ against α-glucosidase: 0.25 μg/mL	[29,30]
	*Callyspongia* *samarensis*	-	EC_50_ 14.47 μg/mL (AMPK Activation)	[31]
	*Callyspongia* sp.	β-Sitosterol	Activation of GLUT-4 and insulin receptors	[32]
Antihypercholesterolemic	*Callyspongia* sp.	Callyspongiamide A	IC_50_ against SOAT-1 and SOAT-2: 0.78 ± 0.19 and 2.8 ± 0.72 μM	[5]
		Callyspongiamide B	IC_50_ against SOAT-1 and SOAT-2: 1.2 ± 0.31 and 2.4 ± 0.96 μM	
		Disamide A	IC_50_ against SOAT-1 and SOAT-2: 5.2 ± 0.93 and 4.2 ± 0.76 μM	
Antihypertensive	*Callyspongia* *diffusa*	Callypyrone A	IC_50_ against Angiotensin I-converting enzyme (ACE): 0.48 mM	[33]
		Callypyrone B	IC_50_ against ACE: 0.57 mM	
Anti-inflammatory	*Callyspongia crassa*	-	61.47% inhibition of protein denaturation	[34]
	*Callyspongia* sp.	-	97% inhibition of hemolysis (at a dose of 3200 ppm)	[35]
	*Callyspongia* sp.	Cyclo[L-Hyp-L-Ala]	Increase secretion of IL-10 (J774A.1 cells) by 1.65-fold	[36]
		Cyclo[L-Pro-Gly]	Increase secretion of IL-10 (J774A.1 cells) 1.29-fold	
		Cyclo[L-Pro-Phe]	Increase secretion of IL-10 (J774A.1 cells) 1.54-fold	
		Cyclo[L-Pro-Ala]	Increased secretion of IL-10 (J774A. 1 cells) 1.56-fold	
	*Callyspongia* sp.	β-Sitosterol	ED_50_ 155.6 (mg/kg/ip) on adrenal pituitary axis 54% of inflammatory effect at dose 320 mg/kg (p.o.)	[37]
	*Callyspongia* *siphonella*	Callysterol	19.5 ± 7.3 mL (Edema volume)	[38]
Antifungal	*Callyspongia aff. implexa*	Gelliusterol E	IC_50_ against *Chlamydia trachomatis*: 2.34 ± 0.22 µM (No inclusion at a concentration of 40 μM)	[39]
	*Callyspongia* *aerizusa*	Callyaerin A	*Chlamydia albican* inhibition with a zone of inhibition of 25–30 mm	[6]
		Callyaerin B	*Chlamydia albican* inhibition with a zone of inhibition of 15 mm	
		Callyaerin E	*Chlamydia albican* inhibition with a zone of inhibition of 20 mm	
	*Callyspongia* sp.	β-Sitosterol	Average inhibition diameter against *Fusarium* spp.: 10 mm	[39,40]
	*Callyspongia* sp.	(-)-Siphonodiol	MIC against *Trichophyton asteroides*: 25.0 μg/mL	[41]
	*Callyspongia* *fibrosa*	4-hydroxybenzoic acid	Antifungal against *Ganoderma boninense*	[42,43]
Cytotoxicity against cancer cell lines	*Callyspongia* *siphonella*	-	IC_50_ against:	[34]
			Caco-2 cell line: 5.57 μg/mL MCF-7 cell line: 1.39 μg/mL	
		Neviotine-C	IC_50_ against:	[44]
			PC-3 cell line: 53.6 ± 0.17 μM A549 cell line: 87.2 ± 1.34 μM MCF-7 cell line: 45.5 ± 0.06 μM	
		Neviotine A	IC_50_ against:	
			PC-3 cell line: 71.2 ± 0.34 μM A549 cell line: 76.3 ± 0.35 μM MCF-7 cell line: 46.3 ± 0.06 μM	
			IC_50_ against:	[45]
			MCF-7 cell line: 12.3 ± 0.7 μg/mL HepG-2 cell line: 11.8 ± 1.2 μg/mL	
		Sipholenol-A	IC_50_ against:	[44]
			PC-3 cell line: 7.9 ± 0.12 μM A549 cell line: 8.9 ± 0.01 μM MCF-7 cell line: 56.3 ± 0.17 μM	
			IC_50_ against:	[45]
			MCF-7 cell line: 19.2 ± 0.6 μg/mL HepG-2 cell line: 9.6 ± 0.8 μg/mL	
		Sipholenone A	IC_50_ against:	[44]
			PC-3 cell line: 53.9 ± 0.25 μM A549 cell line: 24.8 ± 0.22 μM MCF-7 cell line: 36.2 ± 0.13 μM	
			IC_50_ against:	[45]
			MCF-7 cell line: 3 ± 0.4 μg/mL HepG-2 cell line: 2.8 ± 0.4 μg/mL	
		Sipholenol L	IC_50_ against:	
			MCF-7 cell line: 4.0 ± 0.22 μg/mL HepG-2 cell line: 18.7 ± 0.9 μg/mL	
	*Callyspongia crassa*	-	IC_50_ against:	[34]
			Caco-2 cell line: 13.05 μg/mL MCF-7 cell line: 9.47 μg/mL	
	*Callyspongia* sp.	Callyspongiolide	IC_50_ against:	[46]
			L5178Y cell line: 320 nM Jurkat J16 T cell line: 70 nM Ramos B lymphocyte cell line: 60 nM	
	*Callyspongia* sp.	Callypeptide A	GI_50_ against:	[47]
			MDA-MB-231 cell line: 29 μM HT-29 cell line: 30 μM A549 cell line: 18.5 μM	
	*Callyspongia* sp.	Callyazepin	IC_50_ against:	[48]
			K562 cell line: 7.4 μM A549 cell line: 3.0 μM	
		(3R)-methylazacyclodecane	IC_50_ against:	
			K562 cell line: 3.2 μM A549 cell line: 3.8 μM	
	*Callyspongia* sp. (CMB-01152)	Hymenialdisine	IC_50_ against:	[49]
			SW620 cell line: 3.1 μM KB-3-1: 2.0 μM	
	*Callyspongia schulzei*	-	IC_50_ against:	[50]
			HT-29 cell line: 35.57 ± 0.87 μg/mL T47D cell line: 37.98 ± 2.12 μg/mL Casky tumor cell line: 63.20 ± 0.76 μg/mL	
	*Callyspongia* *aerizusa*	Callyaerin E	IC_50_ against L5178Y cell line: 0.39 μM	[6]
		Callyaerin H	IC_50_ against L5178Y cell line: 0.48 μM	
	*Callyspongia* *truncata*	Callystatin	IC_50_ against KB cell line: 0.01 μg/mL	[51]
		-	Further research: IC_50_ against:	
			KB cell line: 10 pg/mL L1210 cell line: 20 pg/ml	
	*Callyspongia* sp.	(−)-(3R,18R) alcohol	IC_50_ against TR-LE cell line: 0.11 μM	[52]
		(+)-(3S,18S)	IC_50_ against TR-LE cell line: 0.47 μM	
	*Callyspongia* sp.	Siphonodiol	IC_50_ against HL-60 cell line: 6.5 μg/mL	[53]
		Callyspongidiol	IC_50_ against HL-60 cell line: 2.8 μg/mL	
		14,15-dihydrosphonodiol	IC_50_ against HL-60 cell line: 6.5 μg/mL	
	*Callyspongia* sp.	Callyspongenols A	IC_50_ against:	[54]
			P388 cell line: 2.2 μg/mL HeLa cell line: 4.5 μg/mL	
		Callyspongenols B	IC_50_ against:	
			P388 cell line: 10 μg/mL HeLa cell line: 10 μg/ mL	
		Callyspongenols C	IC_50_ against:	
			P388 cell line: 2.2 μg/mL HeLa cell line: 3.9 μg/mL	
		Callyspongenols D	IC_50_ against:	
			P388 cell line: 0.4 μg/mL HeLa cell line: 0.066 μg/mL	
	*Callyspongia* *fistularis*	Callyspongamide A	IC_50_ against HeLa cell line: 4.1 μg/mL	[55]
	*Callyspongia* sp.	Alkupikanye E	IC_50_: 5 μg/mL	[56]
		Alkupikanye F	IC_50_: 10 μg/mL	
	*Callyspongia* sp.	-	IC_50_: 2 μg/mL against NIH3T3 cells transfected with the human EGF receptor	[57]
		8-Bromooctyl tert-butyldimethylsilyl ether (fraction, *n* = 3) 9-(3-Pyridyl)nonyl alcohol (fraction, *n* = 3)	IC_50_: 1.3 μg/mL against NIH3T3 cells transfected with human EGF receptor gene	
	*Callyspongia* sp.	Akaterpin	IC_50_ against PI-PLC: 0.5 μg/mL	[58]
	*Callyspongia* *aerizusa*	-	IC_50_ against:	[59]
			A549 cell line: 9.38 μg/mL TE-8 cell line: 3.12 μg/mL HEP G2 cell line: 10.62 μg/mL MIA PaCa-2 cell line: 10.72 μg/mL	
Antimicrobial	*Callyspongia crassa*	-	LC_50_ against:	[60]
			*Staphylococcus aureus*: 215.2 ± 32.9 μg/mL *Bacillus subtilis*: 18.2 ± 3.56 μg/mL	
	*Callyspongia* *siphonella*	Siphonocholin	MIC against *Pseudomonas aeruginosa*: 64 μg/mL	[61]
		Sipholenol L	Inhibition against:	[45]
			*Staphylococcus aureus* (Zone of inhibition: 12.3 ± 0.72 mm) *Bacillus subtitus* (Zone of inhibition: 14.5 ± 0.72 mm)	
		Neviotine A	Inhibition against:	
			*Staphylococcus aureus* (Zone of inhibition: 14.1 ± 0.72 mm) *Bacillus subtitus* (Zone of inhibition: 17.2 ± 0.58 mm) *Escherichia coli* (Zone of inhibition 12.7 ± 0.58 mm)	
		Sipholenone A	Inhibition against:	
			*Staphylococcus aureus* (Zone of inhibition: 8.2 ± 0.72 mm) *Bacillus subtitus* (Zone of inhibition: 2.4 ± 0.58 mm) *Escherichia coli* (Zone of inhibition 5.4 ± 0.58 mm)	
	*Callyspongia aerizusa*	Callyaerin A	Inhibition against:	[6]
			*Escherichia coli* (moderate) with zone of inhibition: 10–15 mm *Staphylococcus aureus* (mild) with zone of inhibition: 9 mm	
		Callyaerin E	Inhibition against:	
			*Bacillus subtilis* (potent) with zone of inhibition: 15–17 mm *Escherichia coli* (mild) with zone of inhibition: 9–11 mm *Staphylococcus aureus* (mild) with zone of inhibition: 9–10 mm	
Antioxidant	*Callyspongia crassa*	-	Percentage of inhibition: 58.1% at 671 μg/mL	[34]
	*Callyspongia* *aerizusa*	-	Percentage of inhibition 56.6% at 0.5 μg/mL, 57.2% at 0.6 μg/mL, and 58.4% at 0.7 μg/mL	[62]
Antiparasitic	*Callyspongia* sp.	Isoakaterpine	IC_50_ against adenosine phosphoribosyltransferase of *Leishmania* spp: 1.05 μM	[11]
Antiallergic	*Callyspongia* sp.	3-(2-(4-hydroxyphenyl)-2-oxoethyl)-5,6-dihydropyridine-2(1H)-one	IC_50_ againts RBL-2H3: 18.7 ± 6.7 μM	[63,64]
Antituberculosis	*Callyspongia* *aerizusa*	Callyaerin A	MIC_90_ against *Mycobacterium tuberculosis*: 2 μM	[64]
		Callyaerin B	MIC_90_ against *Mycobacterium tuberculosis*: 5 μM	
Antiviral	*Callyspongia crassa*	-	85.3% against hepatitis A virus (MIC 9.765 μg/mL)	[34]
	*Callyspongia* *siphonella*	-	83.7% against hepatitis A virus (MIC 0.625 μg/mL)	
Immunomodulatory	*Callyspongia* sp.	Niphatoxin C	IC_50_ P2X_7_ antagonism: 11.5 μM	[65]
	*Callyspongia* sp.	Siphonodiol	Increases IL-12p70 secretion in LPS-primed DCs Modulates dendritic cell function for T1 cell proliferation	[66]
	*Callyspongia* sp.	Callyspongidiol	Modulates dendritic cell function for T1 cell proliferation	[53]
		14,15-dihydrosphonodiol		
	*Callyspongia* sp.	β-Sitosterol	Modulates dendritic cell activity and increases peripheral blood mononuclear cell viability	[67]
	*Callyspongia* sp.	-	Increase levels of IFN-γ and TNF-α (Wistar strain mice) at extract doses of 300 mg/kg and 400 mg/kg	[68]
Antineurodegenerative	*Callyspongia* *samarensis*	-	IC_50_ against β-secretase: 99.82 μg/mL	[31]
	*Callyspongia* sp.	Hymenialdisine	IC_50_ against:	[49]
			GSK3β: 0.07 μM CK5.p25: 0.16 μM CK1δ: 0.03 μM	
Antiosteoporotic	*Callyspongia* *siphonella*	Neviotine D	IC_50_ against RANKL: 12.8 μM	[69]
		Neviotine A	IC_50_ against RANKL: 32.8 μM	

IC_50_: Half maximal inhibitory concentration; EC_50_: Half maximal effective concentration; ED_50_: Median effective dose; MIC: Minimum inhibitory concentration; GI_50_: Half maximal growth inhibition; LC_50_: Median Lethal Concentration.
